# Intravenous immunoglobulin prevents peripheral liver transduction of intrathecally delivered AAV vectors

**DOI:** 10.1016/j.omtm.2022.09.017

**Published:** 2022-10-04

**Authors:** Makoto Horiuchi, Christian J. Hinderer, Jenny A. Greig, Cecilia Dyer, Elizabeth L. Buza, Peter Bell, Jessica A. Chichester, Peter M. Hayashi, Hanying Yan, Tamara Goode, James M. Wilson

**Affiliations:** 1 Gene Therapy Program, Department of Medicine, Perelman School of Medicine, University of Pennsylvania, Philadelphia, PA 19104, USA

**Keywords:** AAV, intrathecal, intravenous immunoglobulin, neutralizing antibodies, central nervous system

## Abstract

Gene therapy using neurotropic adeno-associated virus vectors represents an emerging solution for genetic disorders affecting the central nervous system. The first approved central nervous system-targeting adeno-associated virus gene therapy, Zolgensma®, for treating spinal muscular atrophy is administered intravenously at high doses that cause liver-associated adverse events in 20%–30% of patients. Intrathecal routes of vector administration, such as the intra-cisterna magna route, provide efficient gene transduction to central nervous system cells while reducing off-target liver transduction. However, significant levels of liver transduction often occur upon intra-cisterna magna vector delivery in preclinical studies. Using vectors expressing monoclonal antibody transgenes, we examined whether passive transfer of adeno-associated virus-neutralizing antibodies as intravenous immunoglobulin before intrathecal adeno-associated virus delivery improved the safety of viral gene therapy targeting the central nervous system in mice and nonhuman primates. We used intracerebroventricular and intra-cisterna magna routes for vector administration to mice and nonhuman primates, respectively, and evaluated transgene expression and vector genome distribution. Our data indicate that pretreatment with intravenous immunoglobulin significantly reduced gene transduction to the liver and other peripheral organs but not to the central nervous system in both species. With further refinement, this method may improve the safety of adeno-associated virus-based, central nervous system-targeting gene therapies in clinical settings.

## Graphical abstract

**Figure:**
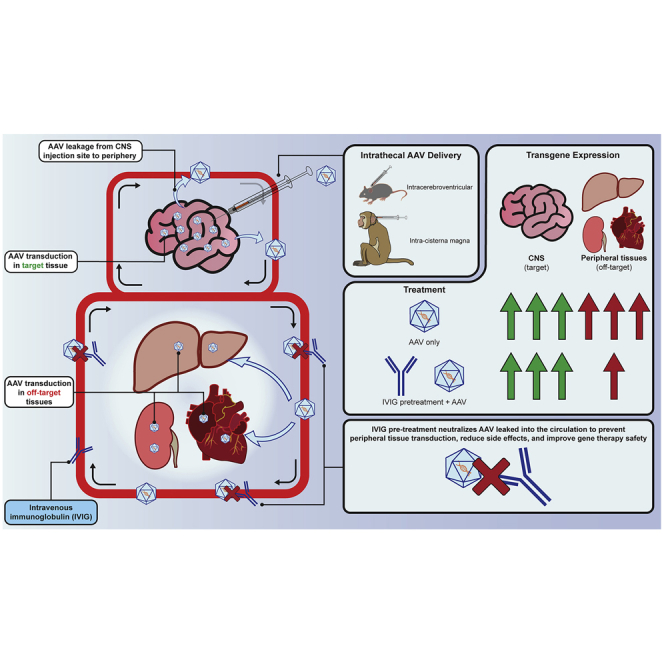

## Teaser

Off-target transduction during AAV gene therapy is associated with adverse side effects. Horiuchi et al. examined whether pretreatment with AAV-neutralizing antibodies prevented off-target transduction in mice and nonhuman primates receiving CNS-targeted gene therapy. Intravenous immunoglobulin pretreatment significantly reduced transduction of the liver and other peripheral organs but not the CNS.

## Introduction

Many genetic disorders negatively affect the normal development and function of the central nervous system (CNS). Gene therapy using neurotropic adeno-associated virus (AAV) vectors such as AAV9 represents an emerging real-world solution for such diseases. Zolgensma®, an AAV9 vector expressing functional SMN1, has received approval in the United States and other countries as the first CNS-targeting gene therapy for patients with spinal muscular atrophy (SMA). Zolgensma® is administered intravenously and involves the use of very high doses to effectively reach target cells in the spinal cord. Our previous studies showed that high-dose intravenous AAV administration causes severe toxicity in nonhuman primates (NHPs) that is characterized by thrombocytopenia and acute liver injury.^1^ Similar toxicity has been reported in the clinical setting: 20%–30% of patients treated with Zolgensma® exhibit liver-associated adverse events, with a few serious cases requiring additional treatment.^2^

Intrathecal delivery of AAV vectors allows effective transduction of neurons, astrocytes, and ependymal cells, as the blood-brain barrier is bypassed. More specifically, intra-cisterna magna (ICM) administration of AAV gene therapy enables more widespread transgene expression throughout the CNS of NHPs than lumbar puncture.^3^^,^^4^ A study of image-guided intrathecal AAV delivery suggested that ICM delivery is a more reliable technique with less vector leakage from the intrathecal space compared to lumbar puncture.^5^ Delivering AAV gene therapy via the ICM route potentially reduces the total vector dose required to provide a therapeutic level of transgene expression in the CNS, thereby decreasing off-target liver transduction, compared with intravenous vector administration. However, a significant portion of injected vectors can leak and/or diffuse from the intrathecal space during ICM AAV administration and result in a considerable level of liver transduction in large animals.^6^

Methods for reducing off-target liver transduction are crucial for improving the precision of CNS-targeting AAV gene therapy. Emerging capsid engineering approaches, such as directed evolution, have provided promising preclinical data on novel AAVs that exhibit liver de-targeting and increased CNS selectivity properties upon intravenous administration for CNS diseases.^7^^,^^8^ We and others previously observed that gene transduction to the CNS was not affected by pre-existing anti-AAV neutralizing antibodies (NAbs), while liver transduction decreased significantly upon intrathecal vector administration in dogs and NHPs.^9^, ^10^, ^11^ On the basis of these observations, we hypothesized that the passive transfer of AAV NAbs may prevent transduction to peripheral organs such as the liver with minimal impact on CNS transduction upon ICM AAV delivery in patients without pre-existing NAbs. To test this possibility, we measured tissue-specific transgene expression in mice and NHPs pretreated with intravenous immunoglobulin (IVIG) - which provides NAbs - before intrathecal AAV administration. IVIG comprises purified human IgG pooled from a healthy human population and is commonly used as a therapeutic for a wide range of immune-related diseases. Because of the high prevalence of AAV NAbs in the human population, IVIG has a high NAb titer.^12^ To investigate and evaluate the potential utility of pre-existing NAbs in AAV gene therapy, we used IVIG administration (rather than NAb-positive animals) as an established, defined clinical intervention,^13^ which may have translational potential in the context of gene therapy. Our data indicate that pretreatment with IVIG significantly reduced vector transduction in the liver and other peripheral organs but not in the CNS in both species. Thus, IVIG pretreatment followed by ICM vector administration may represent a strategy for preventing off-target effects underpinned by non-CNS transduction upon intrathecal AAV gene therapy that can be easily translated to the clinic.

## Results

### ICV AAV treatment results in peripheral transgene expression in addition to CNS expression in mice

**Figure 1:**
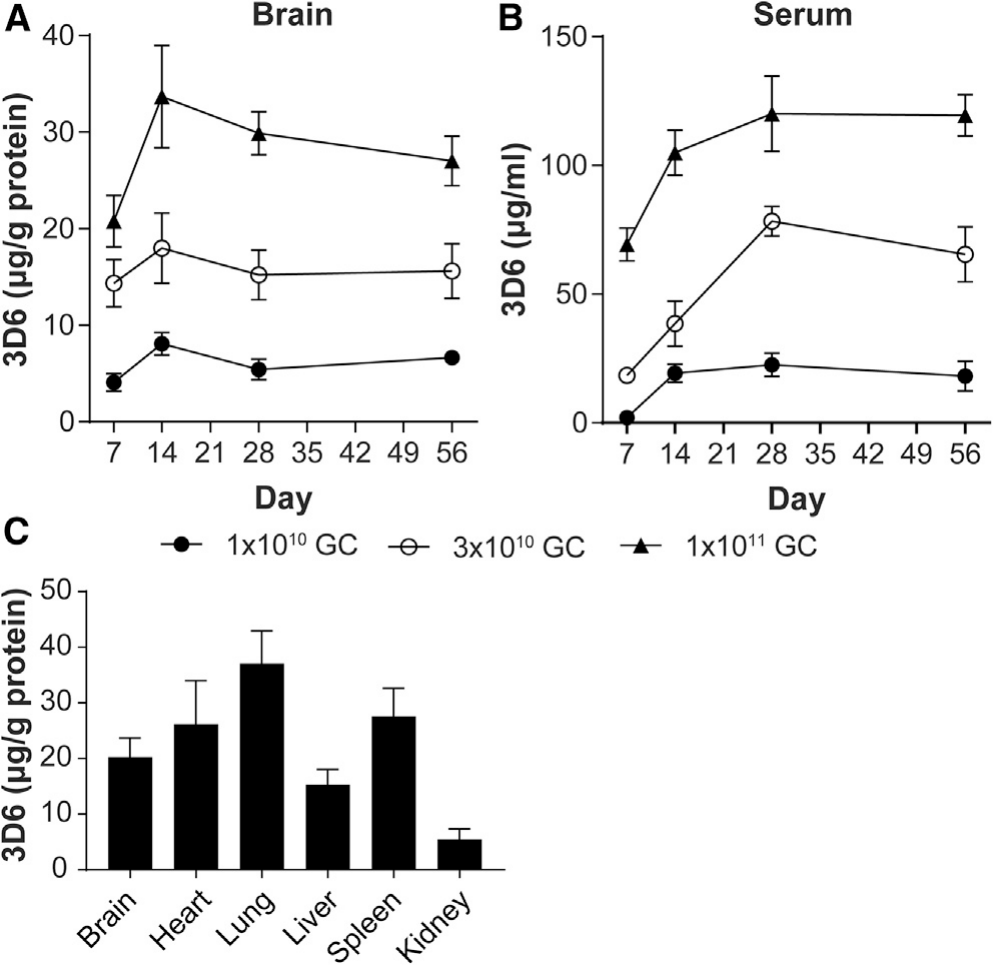
Pharmacokinetics and protein biodistribution of transgene product 3D6 mouse monoclonal antibody in mice after an ICV dose of AAVhu68.CB7.3D6 Mice received the vector via unilateral ICV administration at the indicated doses, and 3D6 expression in brain homogenates (A) and serum (B) was measured using an antigen-binding assay at 7, 14, 28, and 56 days post-AAV treatment. 3D6 was measured in homogenates of selected organs from mice treated with 1 × 10^10^ GC at 56 days post-AAV administration (C). Values are presented as mean ± SEM.

We administered an AAVhu68 vector expressing a mouse monoclonal antibody (3D6) via unilateral intracerebroventricular (ICV) injection to wild-type mice at three doses. Mice were euthanized on days 7, 14, 28, and 56, and 3D6 concentrations were measured in the brain and serum. 3D6 levels showed a dose-dependent response in both the brain and serum. In the brain, 3D6 peaked at day 14 and remained at approximately 80% of the peak expression through day 56 (Figure 1A). In serum, 3D6 accumulated relatively slowly and reached a plateau by day 28 (Figure 1B). Sustained high levels of serum 3D6 expression suggest the transduction of peripheral organs such as the liver and skeletal muscle. Consistent with this finding, we also detected 3D6 protein expression by enzyme-linked immunosorbent assay (ELISA) in peripheral organs, including the heart and liver, in mice treated with ICV AAV at a dose of 3 × 10^10^ genome copies (GC) (Figure 1C). We selected this intermediate dose, 3 × 10^10^ GC/mouse, for further studies.

### Passive NAb transfer prevents peripheral organ transduction while allowing CNS transduction of AAVhu68.3D6 in mice

**Figure 2:**
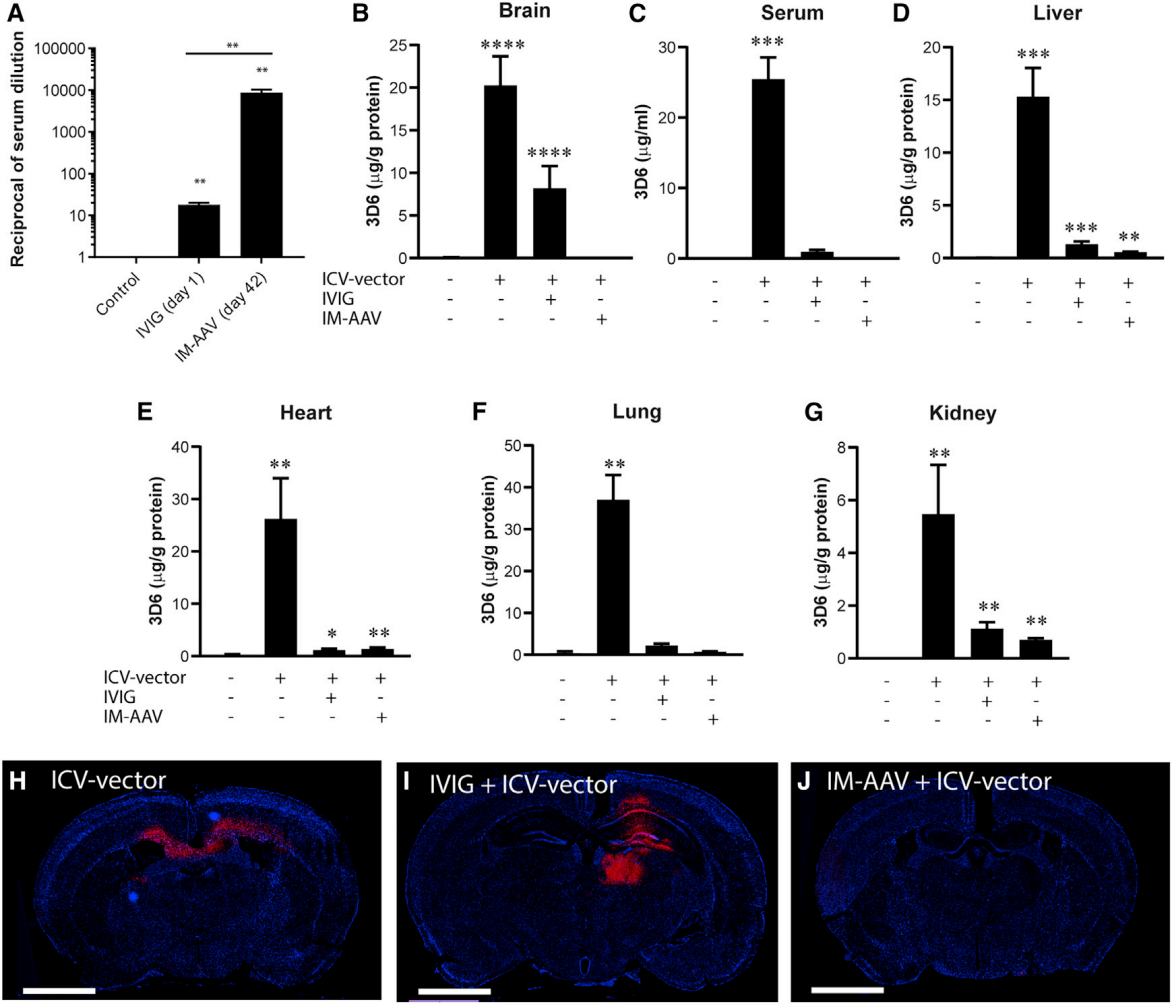
Passive NAb transfer prevents peripheral organ transduction while allowing CNS transduction of AAVhu68.3D6 in mice (A) Average AAVhu68 NAb titers in the reciprocal of serum dilution. Mice pretreated with IVIG received AAV on day 1 after IVIG (IVIG [day 1]). Mice actively immunized with IM-AAV were injected with ICV-AAV.3D6 at 42 days after IM-AAV (IM-AAV [day 42]). Serum samples from naive mice were used as controls. ∗∗p < 0.01. (B–G) 3D6 expression in mouse tissue homogenates at 56 days post-ICV-vector treatment. 3D6 antigen-binding assay data for brain (B), serum (C), liver (D), heart (E), lung (F), and kidney (G) samples from mice with IVIG pretreatment followed by ICV-AAV.3D6 treatment, mice with IM-AAV followed by ICV-AAV.3D6 treatment, or mice with only ICV-AAV.3D6 treatment. Tissue homogenates from untreated mice were used as a negative control. ∗p < 0.05, ∗∗p < 0.01, ∗∗∗p < 0.005, and ∗∗∗∗p < 0.001 compared with the negative control. Values are presented as mean ± SEM. (H–J) 3D6 *in situ* hybridization for mouse brain sections at 28 days post-ICV-vector administration. Paraffin-embedded coronal brain sections from mice with IVIG pretreatment followed by ICV-AAV.3D6 treatment (IVIG + ICV-vector), mice with IM-AAV followed by ICV-AAV.3D6 treatment (IM-AAV + ICV-vector), and mice with only ICV-AAV.3D6 treatment (ICV-vector) were hybridized with a fluorescent-labeled 3D6-specific probe (red). Nuclei were counterstained with DAPI (blue). Scale bars indicate 2 mm.

To examine the effect of circulating NAbs on intrathecally administered AAV transgene expression, we pretreated mice with IVIG at 0.5 g/kg 24 h before administering ICV-AAVhu68.3D6 (IVIG + ICV-vector). The dose and timing of IVIG were selected from a pilot optimization study that identified an optimal AAVhu68 NAb titer of 1:10 to 1:20 at the time of vector administration. The specific IVIG lot used in this study had a 1:1,280 AAVhu68 NAb titer in 100 mg/mL solution. Mice that had received 1 × 10^11^ GC AAVhu68.CB7.eGFP intramuscularly (IM) 42 days earlier also received ICV-AAV as an active immunization group (IM-AAV + ICV-vector). An AAVhu68 neutralization assay indicated that IVIG infusion resulted in a 1:20 AAVhu68 NAb titer at the time of ICV-vector administration, whereas IM-AAVhu68.eGFP achieved an NAb titer of 1:10,240 (Figure 2A). At 28 days after ICV-AAVhu68.3D6 treatment, 3D6 was detectable in the brain for the IVIG group, although expression levels were approximately 50% lower than those in the brains of mice receiving ICV-vector without IVIG pretreatment (ICV-vector). Expression levels of 3D6 in the serum and peripheral organs (e.g., liver, heart, lung) were less than 10% of those for the ICV-vector controls in IVIG-pretreated mice. The robust reduction of serum 3D6 but retention of high expression in the brains of IVIG-pretreated mice suggests that the majority of serum 3D6 detected in ICV-vector control mice derived from transduced peripheral organs, not the CNS. 3D6 was undetectable or very low in the brain, serum, and other organs in the IM-AAV + ICV-vector group (Figures 2B–2G). *In situ* hybridization analyses confirmed this expression pattern, in which 3D6 was present in the brain parenchyma of the ICV-vector and IVIG groups but not the IM-AAV group (Figures 2H–2J).

### Passive NAb transfer prevents ICM-AAV.mAb transduction to peripheral organs while preserving CNS transduction in NHPs

**Figure 3:**
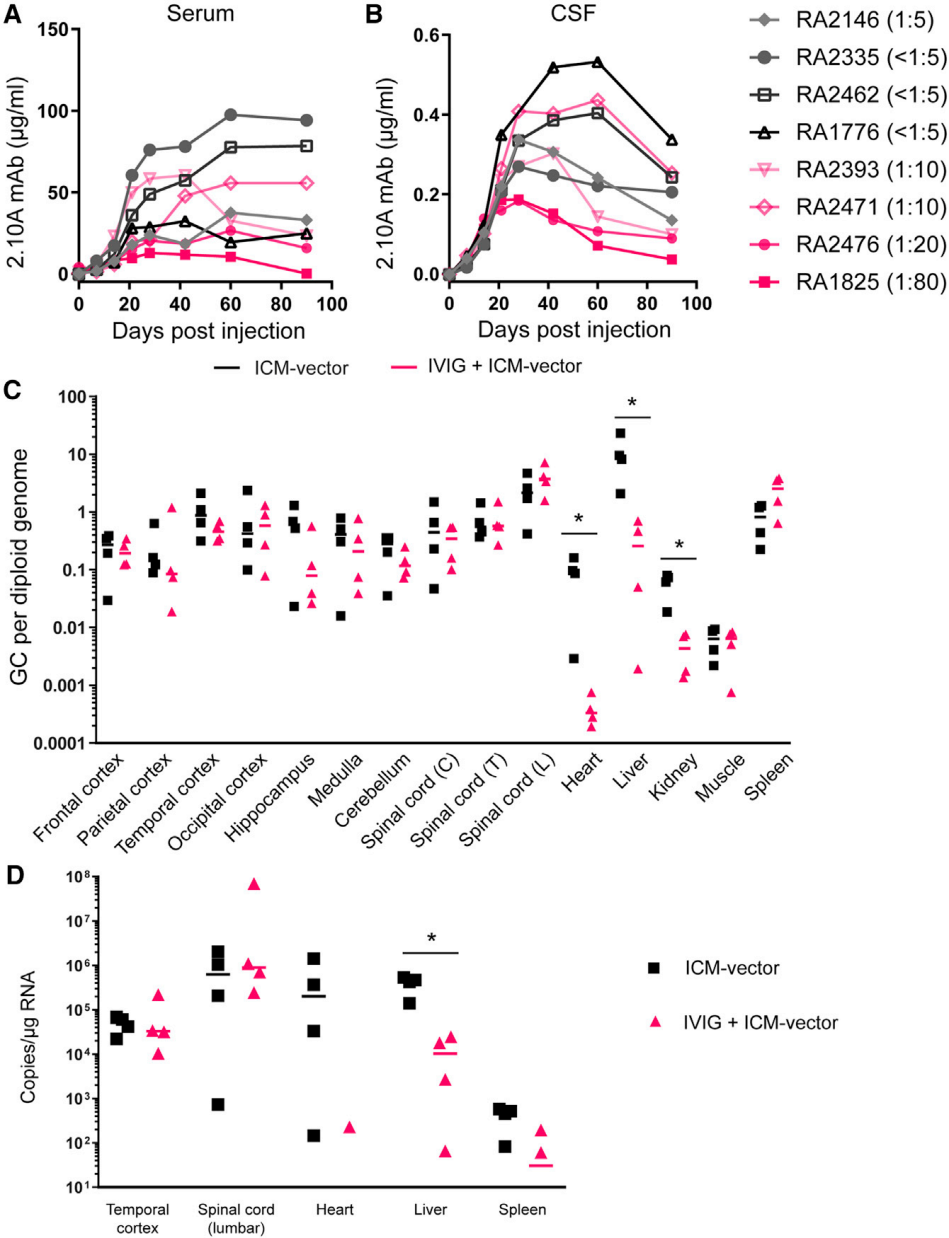
Gene transduction and transgene expression in NHPs treated with ICM-vector expressing 2.10A mAb with or without IVIG pretreatment Average 2.10A mAb concentration in serum (A) and CSF (B) along the course of the study. qPCR with a transgene-specific assay determined vector genome copy numbers (GC) in selected CNS tissue blocks and peripheral tissue blocks (C) and 2.10A mAb mRNA levels in selected CNS tissue blocks and peripheral tissue blocks (D). Data presented as mean ± SEM. ∗p < 0.05.

We used NHPs for further translational studies, as their size and anatomy enable the use of the same image-guided ICM injection technique used in clinical trials. We used these animals to investigate whether IVIG pre-infusion prevented the transduction of peripheral organs while preserving transgene expression in the CNS after ICM-AAV administration. We used a simian monoclonal antibody against simian immunodeficiency virus, 2.10A mAb, as the transgene in this study. All NHPs were prescreened and exhibited AAVhu68 NAb < 1:5. Some NHPs received 0.5 g/kg IVIG at 24 h prior to ICM-AAV.mAb treatment (AAVhu68.CB7.2.10A.mAb at 3 × 10^13^ GC/animal) (group 2; IVIG + ICM-vector group). On the day of ICM-AAV administration (day 0), these pretreated NHPs exhibited NAb titers of 1:10 to 1:80, while control NHPs who did not receive IVIG pretreatment (group 1; ICM-vector group) had low NAb titers of <1:5 or 1:5 (Table 1). We examined the time course of 2.10A mAb expression in serum (Figure 3A) and cerebrospinal fluid (CSF) (Figure 3B) for 88–91 days post-vector administration (i.e., when all NHPs underwent necropsy). In the ICM-vector group, two NHPs with NAb titers <1:5 showed robust and sustained 2.10A mAb expression in serum, whereas the other two NHPs (including one with an increased NAb titer of 1:5 at day 0) showed relatively lower serum expression. In the IVIG + ICM-vector group, serum 2.10A mAb expression varied according to the NAb titer at day 0. Two NHPs with NAb titers of 1:20 and 1:80 at day 0, respectively, showed reduced serum expression, whereas those with NAb titers of 1:10 maintained higher expression levels (Figure 3A). Area under the curve (AUC) analysis highlighted a strong inverse correlation with NAb titer at day 0 in the IVIG + ICM-vector group (Figure S1A). In CSF, the 2.10A mAb level increased similarly among all NHPs during the first 21 days but varied at later time points; NHPs with higher day 0 NAb titers showed relatively lower CSF expression levels. It is known that a small portion (0.1%) of circulating IgG enters CSF by an unknown mechanism.^14^ The variable CSF level achieved at later time points can therefore be explained, at least in part, by the differential contribution of serum-derived 2.10A mAb among animals. AUC analysis showed a moderate inverse correlation with NAb titer at day 0 in the IVIG + ICM-vector group (Figures 3B and S1B).

**Table 1:** Details of the NHP study groups and AAVhu68 NAb titers at day 0

Group	Treatment	Animal ID	Sex	Body weight at baseline (kg)	AAVhu68 NAb titer at baseline	AAVhu68 NAb titer at day 0 (following IVIG administration for group 2)
1	AAVhu68.CB7.2.10A.mAb (3 × 10^13^ GC/animal, ICM, day 0)	RA2146	F	7.2	<1:5	1:5
		RA2335	M	5.7	<1:5	<1:5
		RA2462	M	7.9	<1:5	<1:5
		RA1776	M	10.1	<1:5	<1:5
2	IVIG (0.5 g/kg, IV, day −1) AAVhu68.CB7.2.10A.mAb (3 × 10^13^ GC/animal, ICM, day 0)	RA2393	F	5.9	<1:5	1:10
		RA2471	M	6.3	<1:5	1:10
		RA2476	M	5.7	<1:5	1:20
		RA1825	F	6.2	<1:5	1:80

All NHPs were prescreened and exhibited baseline NAb levels <1:5 prior to study commencement. Following IVIG administration, there were detectable AAVhu68 NAbs.

GC, genome copies; ICM, intra-cisterna magna; IV, intravenous; IVIG, intravenous immunoglobulin; NAb, neutralizing antibody.

Vector genome biodistribution analysis on tissue samples collected at necropsy indicated that AAV transduction occurred throughout the CNS at equivalent levels in both the ICM and ICM + IVIG groups, but transduction was significantly reduced in the heart, liver, and kidney in NHPs pretreated with IVIG (Figure 3C). Individual data show that transduction of some peripheral organs (Figure S2), including the liver, heart, and kidney, but not the CNS (Figure S3), was inversely correlated to NAb titers at day 0. Among the peripheral organs examined, skeletal muscle failed to show such a correlation, likely because of its overall low transduction with an ICM-administered vector. On the contrary, the spleen exhibited a positive correlation, which is consistent with our previous finding in pre-existing NAb+ NHPs administered intravenous vector and suggests that vector may be redirected to this off-target tissue in an antibody-mediated process.^15^ NHPs with an NAb titer of 1:10 at day 0 showed a transduction decrease of approximately 30-fold compared with naive controls, while those with NAb titers greater than 1:20 showed a greater reduction of 300-fold. Moreover, qRT-PCR analyses highlighted that 2.10A mAb expression in the liver, but not in the CNS, was significantly lower in NHPs pretreated with IVIG (Figure 3D). These results are consistent with the hypothesis that IVIG pre-infusion provides anti-AAV NAbs that limit vector transduction to peripheral organs, particularly the liver, while preserving CNS transduction after ICM-AAV treatment.

**Figure 4:**
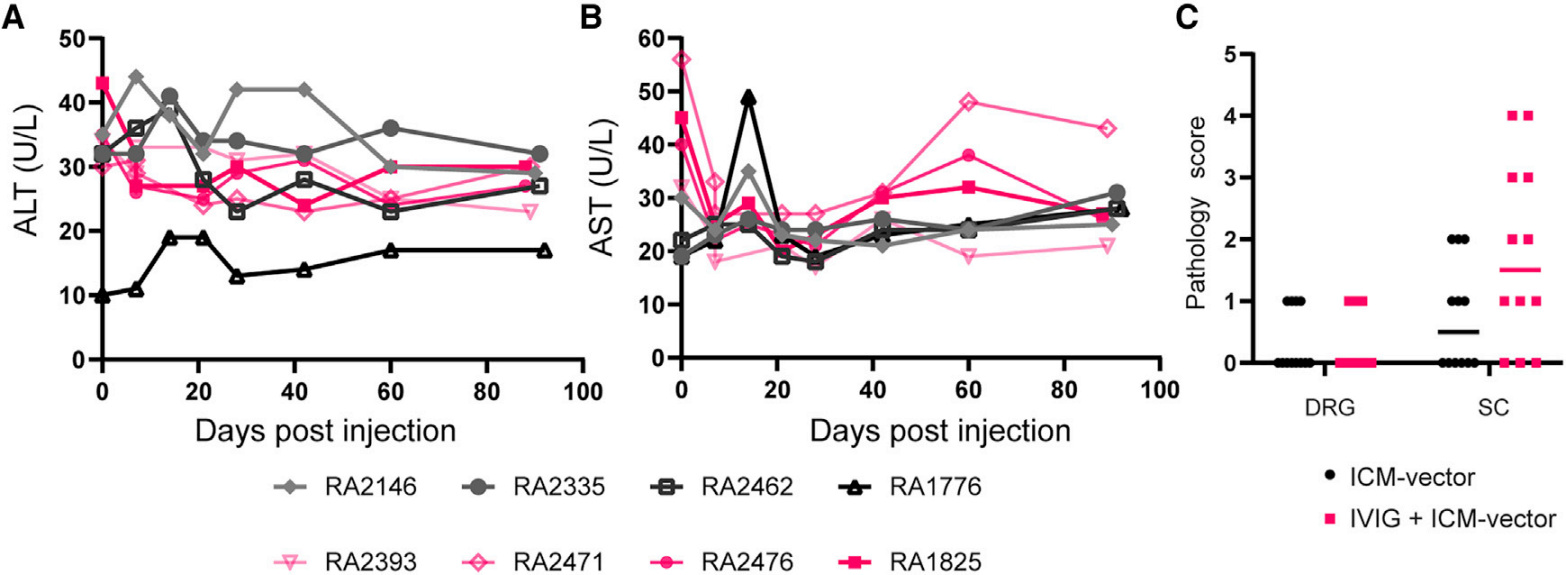
Blood liver enzymes and DRG pathology scores in NHPs Levels of liver enzymes, ALT (A) and AST (B), in NHPs treated with ICM-vector only (RA2146, RA2335, RA2463, and RA1776) and in NHPs receiving IVIG + ICM-vector (RA2393, RA2471, RA2476, and RA1825). Summary of DRG pathology scores (C) for DRG and spinal cord (SC) sections from the NHPs used in this study. Horizontal bars indicate medians of scores.

**Figure 5:**
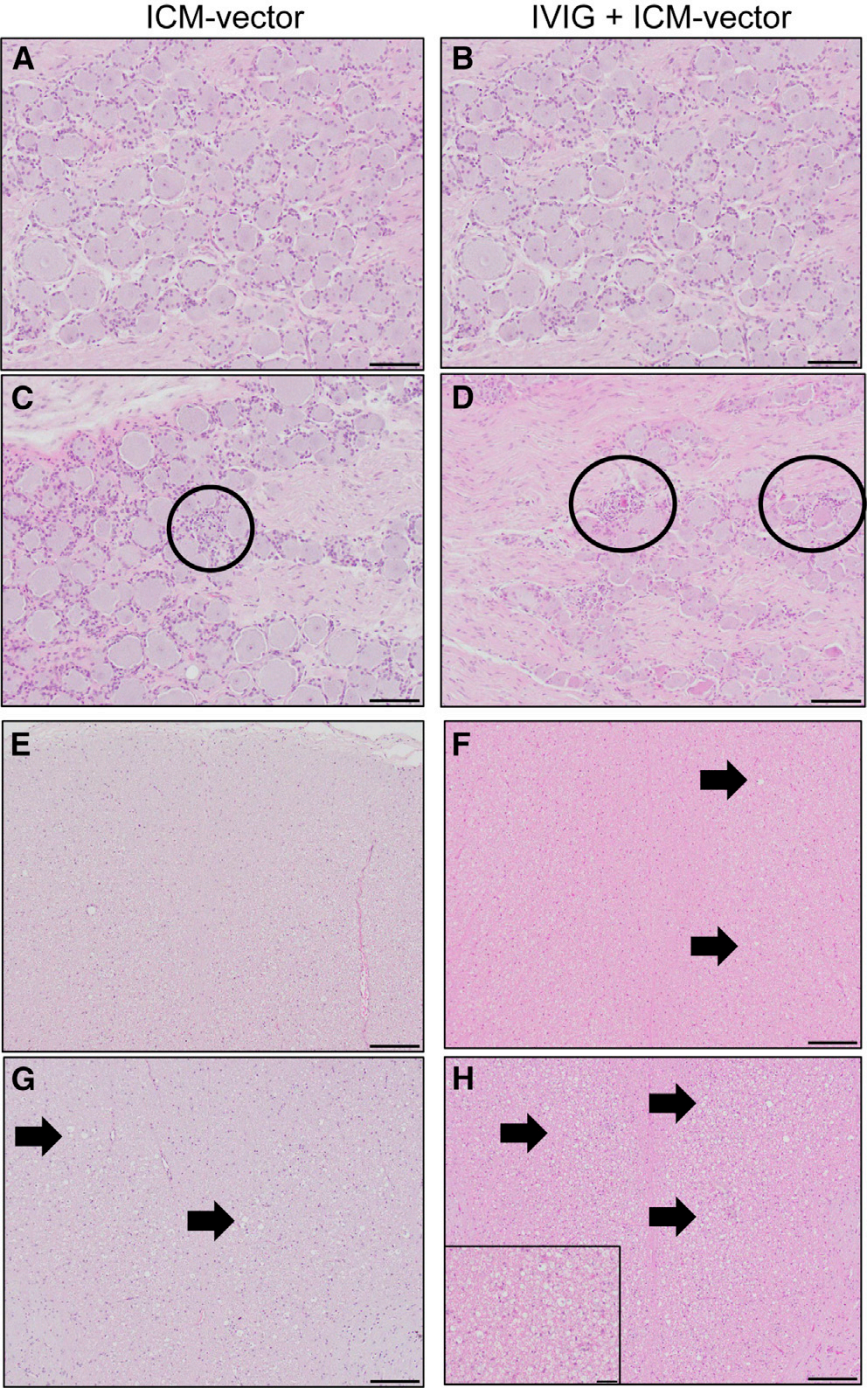
DRG and spinal cord pathology Microscope images depicting severity range of DRG toxicity across ICM-vector and IVIG + ICM-vector groups at necropsy characterized by varying stages of DRG neuronal degeneration and/or necrosis (circles) with associated mononuclear cell infiltrates and secondary axonal degeneration in ascending sensory (dorsal) white matter spinal cord tracts. DRG lesions ranged from normal (A and B) to grade 1 (minimal; C and D) severity. Axonal degeneration in the dorsal white matter spinal cord tracts ranged from normal (E) to grade 1 (minimal; F) to grade 2 (mild; G) to grade 3 (moderate, not shown) to grade 4 (marked; H) severity, characterized by swollen myelin sheaths with axonal debris (arrows) and myelomacrophages (inset). Hematoxylin and eosin; DRG scale bar, 100 μm; spinal cord scale bar, 200 μm; inset scale bar, 50 μm.

Blood alanine transaminase (ALT) and aspartate transaminase (AST) levels remained within normal ranges during the study in both the ICM and ICM + IVIG groups for the vector dose used in this study (Figures 4A and 4B). Blood cell analysis demonstrated that platelet counts were within the normal range for all NHPs throughout the study, except for one NHP in the IVIG group, RA2476 (NAb titer 1:20 at day 0). RA2476 showed a low platelet count (143,000 count/μL) at day 7, but this went back to normal by day 14. There were no overall white or red blood cell count deviations from the baseline in any animals, including RA2476. White blood cell counts fluctuated in some NHPs but returned to baseline levels (Figure S4). Histopathology analyses of dorsal root ganglia (DRG) were performed as described previously.^16^ Analysis of pathology scores from all study animals indicated there were no significant differences in AAV-associated DRG pathology levels between groups (Figure 4C). DRG pathology ranged from normal (Figures 5A and 5B) to grade 1 on the basis of the extent of DRG neuronal degeneration and/or necrosis (Figures 5C and 5D, circles) for both groups. Analysis of axonal degeneration in the dorsal spinal white matter showed that more swollen myelin sheaths with axonal debris and myelomacrophages were observed in IVIG + ICM-vector group compared with the ICM-vector group (Figures 5E–5H).

## Discussion

The aim of this study was to evaluate whether passive immunization against AAV vectors reduced liver transduction while preserving CNS transduction upon intrathecal administration of AAV gene therapy in mice (via ICV) and NHPs (via ICM). Our results from both animal models suggest that IVIG pretreatment may represent a method for improving the safety of CNS-directed AAV gene therapy by reducing adverse effects associated with liver transduction.

In mice, we compared passive immunity transfer via IVIG against active immunization with an IM-AAV injection. *In situ* hybridization data indicated that brain transduction in the IVIG-pretreated groups were comparable to that in the ICV-vector group in mice. However, 3D6 expression appeared to be reduced in the brains of IVIG + ICV-vector mice compared with the ICV-vector group. This likely reflects different methods used to determine 3D6 expression and vector transduction. As circulating IgG can reportedly incorporate into brain endothelial cells and remain in the tissue even after perfusion,^17^ peripherally expressed 3D6 in the circulation may have elevated 3D6 protein levels in the brains of the ICV-vector group, as determined in mice by ELISA. IVIG pretreatment prevented peripheral transduction, thereby indirectly reducing 3D6 protein levels in mouse brain by a significant extent. As vector genome qPCR alone was used to quantify transduction and expression in NHP brains, this reduced expression pattern was not observed.

The passive transfer of immunity was successful in reducing liver transduction while permitting CNS transduction. IM-AAV treatment resulted in a much higher NAb titer that completely prevented CNS transduction. These data are consistent with the findings of Wang et al.^18^; the inhibition of CNS transduction in mice treated with IM-AAV could be due to the resultant extremely high NAb titer and/or pleiotropic immune responses triggered by active immunization. ICV injection is an invasive procedure that artificially introduces blood components to the CSF, including antibodies.^19^ Although the quantity of antibodies entering CNS is probably limited, a small amount may be sufficient to completely block vector transduction in the context of an extremely high NAb titer. It is known that, unlike in humans, capsid-specific T cells induced by AAV immunization are unable to eliminate transduced hepatocytes with intravenous vector administration in mice.^20^ However, this could be different with ICV-vector administration, which is more invasive and damaging to tissues compared with the intravenous route. We previously demonstrated that ICV, but not ICM, administration induces T cell-mediated encephalitis in unimmunized dogs.^10^ In this context, capsid-specific T cells could eliminate transduced brain cells and also contribute to the undetectable level of 3D6 expression in mice with active immunization.

Our data from NHPs highlight the significant translational potential of IVIG pretreatment to minimize off-target liver transduction (and associated adverse events) in patients undergoing CNS-targeting AAV gene therapy. ICM-vector administration, whose efficacy and safety have been established in NHP preclinical studies, is used in clinical studies for CNS gene therapies to treat lysosomal storage disorders and frontotemporal dementia. IVIG is a well-tolerated biologic that is widely used in the clinic for patients with immunodeficiencies, autoimmune diseases, and cytokine storms.^13^ Passive immunization with IVIG prior to ICM gene therapy could be a viable strategy to improve the safety of current and future CNS-targeting AAV gene therapies if peripheral organ transduction is unwanted. For example, some CNS-targeting gene therapies require a high level of transgene expression mainly in the CNS tissue to achieve their therapeutic effect, such as Zolgensma® for SMA. In this case, off-target peripheral transduction causing liver-associated toxicity should be minimized if not avoided.

Limiting peripheral expression of CNS-targeting transgenes that may cause toxic effects in peripheral organs should be a priority in the development of safe therapies. For instance, a vectored monoclonal antibody against human epidermal growth factor-2 (HER2) is under development for breast cancer brain metastases^21^; the off-target activity of the antibody on HER2 expressed in the heart is associated with heart failure.^22^ IVIG for NHPs resulted in varied NAb titers ranging from 1:10 to 1:80 at day 0. For an ICM-vector dose of 3 × 10^13^ GC/animal, NAb titers up to 1:80 did not negatively affect CNS transduction. In contrast, transduction of peripheral organs, including the liver, was significantly impaired by IVIG-derived NAb titers as low as 1:20. This is consistent with our previous study in NHPs with intravenous administration of AAV8 vector.^15^ The degree of peripheral organ transduction appeared to vary according to the NAb titer at day 0 (Figure S1). These data suggest that higher NAb titers of approximately 1:80 may provide superior protection against adverse liver-associated events caused by off-target peripheral transduction without affecting CNS transduction. However, extremely high NAb titers that can develop as a result of AAV gene therapy (and were observed in mice with active immunization) would likely cause CNS transduction upon ICM vector administration to fail in patients, meaning that repeated AAV gene therapy dosing is unlikely to represent a successful approach. This beneficial effect could be particularly useful in optimizing the safety of CNS-targeting gene therapies employing a high ICM AAV vector dose that may cause further off-target liver transduction. To achieve consistently high NAb titers, further improvement in the IVIG dosing regimen with higher doses may be required.

We used the CNS-tropic vector AAVhu68 in this study. The IVIG strategy explored in this study can be theoretically applied to other capsids. However, a limitation of IVIG relates to the fact that it contains different titers of NAbs against different capsids.^12^ Batch variations in NAb titers may also arise, meaning that extensive batch testing and dose adjustments would be required to achieve an appropriate NAb titer on the day of AAV dosing. This strategy could also be applied to other routes of vector administration (e.g., IM) and therefore potentially improve the safety profile of AAV gene therapies directed at target organs other than the CNS. Capsid engineering studies demonstrated promising preclinical data of novel liver de-targeting vectors for cardiac and musculoskeletal gene transfer in addition to those selective to the CNS.^23^ Similar to intrathecal administration, IM administration in naive animals often results in off-target liver transduction (and can cause adverse effects), which is diminished in NAb-positive animals, while transduction of the targeted skeletal muscle is preserved.^24^

Activation of complement is involved in adverse events such as inflammation and thrombocytopenia in high-dose systemic AAV gene therapy.^25^ It is hypothesized that pre-existing anti-AAV antibodies enhance this process by forming immune complexes with AAV vectors and activating the classic complement pathway.^1^ Although the introduction of NAb using IVIG could increase this risk, delivering AAV via ICM limits peripheral vector loads compared with systemic AAV delivery, which could reduce AAV-antibody interaction and any resultant complement activation. Our blood work analysis shows that one NHP in the IVIG group, RA2476 (which had a NAb titer 1:20 at day 0), exhibited a sign of thrombocytopenia at day 7, without changes in white and red blood cell numbers, whereas this was not displayed by any other NHPs, including RA1825 with NAb = 1:80 at day 0 (Figure S4A). These data make it difficult to conclude whether IVIG contributed to post-AAV complement activation and thrombocytopenia. The DRG pathology finding was unexpected, as IVIG itself is safe and commonly used to treat many diseases and conditions, including Guillain-Barré syndrome, which affects DRG neurons.^26^ Further investigation and monitoring, including that of intravenous vector groups, are required to address this issue as well as other safety concerns.

Collectively, our data indicate that passive NAb transfer by IVIG, a well-established clinical product, reduces off-target liver transduction without affecting CNS transduction when the AAV vector is injected intrathecally. Further refinement of this method has the potential to improve the safety of CNS-targeting AAV gene therapies in the clinical setting.

## Materials and methods

### Vectors

We cloned 3D6 and 2.10A mAb into an expression construct flanked by AAV2 inverted terminal repeats containing a chicken beta-actin promoter with a cytomegalovirus early enhancer, chimeric intron, and rabbit beta-globin poly A sequence. AAVhu68 vectors were generated via triple transfection of HEK293 cells and iodixanol purification, as previously described.^27^

### Animal procedures

All animal procedures were approved by the Institutional Animal Care and Use Committee of the University of Pennsylvania and the Children’s Hospital of Philadelphia.

We purchased 6- to 8-week-old C57BL/6J female mice from the Jackson Laboratory (Ben Harbor, ME; stock #000664) for this study. Privigen (CLS Behring, King of Prussia, PA) was used as IVIG and administered to mice via the tail vein for passive NAb transfer. For active immunization of AAV, IM administration of AAVhu68.CB7.CI.eGFP.WPRE.rBG vector was applied to the gastrocnemius for both sides at 5 × 10^10^ GC in 25 μL per side. We performed ICV injection with a previously described freehand technique.^28^ Mice were euthanized via exsanguination/cardiac perfusion with Dulbecco’s PBS while under deep anesthesia with isoflurane delivered via a facemask at the study endpoint. We purchased eight 4- to 6-year-old rhesus macaques (3 females and 5 males; baseline body weight data in Table 1) from Covance Research Products (Denver, PA). NHP studies were conducted at the University of Pennsylvania, in a U.S. Department of Agriculture (USDA)-registered, Association for Assessment and Accreditation of Laboratory Animal Care (AAALAC)-accredited, and Public Health Service (PHS)-assured vivarium. Animals were housed in stainless steel cages in compliance with the Guide for the Care and Use of Laboratory Animals on a 12 h light-dark cycle. A variety of food treats including fresh produce and manipulanda such as toys and mirrors were provided daily, along with visual and auditory enrichment as part of the standard enrichment process. As the IVIG group, four animals received Privigen at 0.5 g/kg intravenously on study day −1. On day 0, all animals received a single ICM injection of 3 × 10^13^ GC of AAVhu68.CB7.CI.2.10A.mAb.SV40 vector in 1 mL of artificial CSF via fluoroscope image guidance, as previously described.^29^

### Enzyme-linked immunosorbent assay

We measured 3D6 mAb levels in serum or tissue homogenates using sandwich enzyme-linked immunosorbent assay using the antigen amyloid-β 1-42 peptide (ab120301; Abcam, Waltham, MA), and horseradish peroxide (HRP)-conjugated anti-mouse IgG antibody for capture and detection, respectively. For 2.10A mAb, the antigen used as the capture protein and the detection reagents were described previously.^24^ Plates coated with the capture protein were blocked and incubated with diluted samples and then detector reagents, with washing performed between each step. HRP activity in each well was developed with 3,3′,5,5′-tetramethylbenzidine substrate, followed by 450 nm optical density measurements via a Spectramax M3 plate reader (Molecular Devices, San Jose, CA).

### Vector genome and transgene mRNA biodistribution

We snap-froze NHP tissue samples at the time of necropsy and extracted DNA and RNA with the QIAamp DNA Mini Kit (56304; Qiagen, Germantown, MD) and RNeasy Mini Kit (74104; Qiagen), respectively. We measured vector genomes by RT-PCR using the TaqMan assay for SV40 poly A sequence as previously described.^30^ RNA was reverse-transcribed into cDNA using a High-Capacity cDNA Reverse Transcription kit (4368814; Thermo Fisher Scientific, Waltham, MD), and 2.10A mAb cDNA was quantified by RT-PCR with a custom TaqMan assay for the transgene.

### NAb assay

We evaluated NAbs against AAVhu68 using a LacZ reporter gene with luminescence readout as previously described.^12^^,^^31^

### Clinical analysis

Blood liver enzymes - AST and ALT - were measured by laboratory diagnostic service by Antech. DRG pathology was evaluated and scored on DRG and spinal cord sections stained with hematoxylin and eosin, as previously described.^16^

### *In situ* hybridization

Mouse brains were fixed in 10% formalin solution, embedded in paraffin, sectioned, and subjected to *in situ* hybridization. We used the ViewRNA ISH Tissue Assay Kit (Thermo Fisher Scientific) according to the manufacturer’s instructions using a probe specifically designed to codon-optimized 3D6. We detected bound probes via Fast Red precipitation. Sections were counter-stained with 4′,6-diamidino-2-phenylindole (DAPI) to show nuclei.

### Statistical analysis

All quantitative datasets were analyzed using the Wilcoxon rank-sum test using the function “wilcox.test” within R version 4.0.0 (https://cran.r-project.org). We applied the Benjamini-Hochberg procedure to correct for multiple hypothesis testing. Statistical significance was assessed at the 0.05 level after multiple testing adjustments.

## Data Availability

All data discussed in the manuscript are available in the main text or supplemental materials. Complete clinical pathology data can be obtained upon request.

## Supplemental information

Document S1. Figures S1–S 4

Document S2. Article plus supplemental information
