# Intravenous immunoglobulin prevents peripheral liver transduction of intrathecally delivered AAV vectors

**DOI:** 10.1016/j.omtm.2022.11.001

**Published:** 2022-11-12

**Authors:** Makoto Horiuchi, Christian J. Hinderer, Jenny A. Greig, Cecilia Dyer, Elizabeth L. Buza, Peter Bell, Jessica A. Chichester, Peter M. Hayashi, Hanying Yan, Tamara Goode, James M. Wilson

## Main text

(Molecular Therapy: Methods & Clinical Development *27*, 272–280; December 2022)

In the originally published version of this article, the images in Figure 5A and 5B were inadvertently duplicated. This does not affect the findings or interpretation of the study. Figure 5 has been updated with the correct image in 5B; there were no other changes to the figure.

This change has been reflected online, and the authors apologize for this error.

**Figure:**
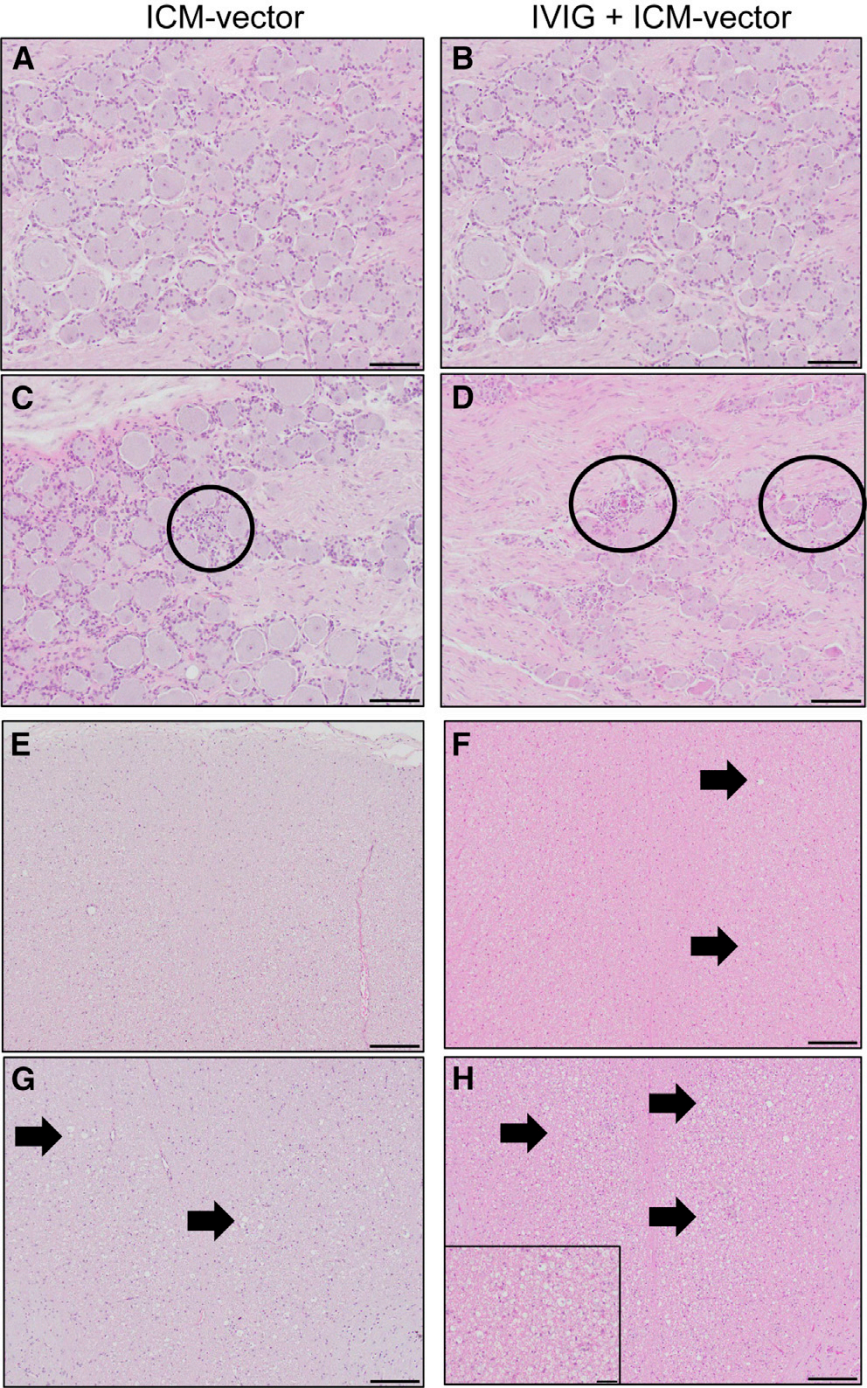
Figure 5. DRG and spinal cord pathology (original)

**Figure:**
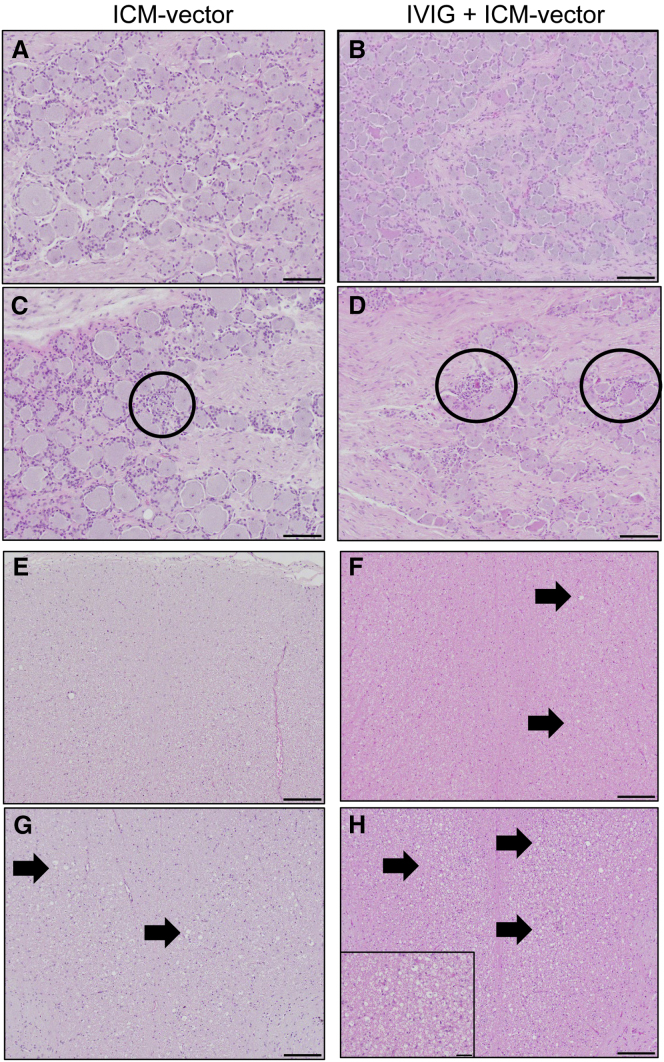
Figure 5. DRG and spinal cord pathology (corrected)

