# Supplementary material for: Intravenous immunoglobulin prevents peripheral liver transduction of intrathecally delivered AAV vectors
Source: Mol Ther Methods Clin Dev. 2022 Oct 4;27:272–80. doi: 10.1016/j.omtm.2022.09.017 (PMC9593247; doi:10.1016/j.omtm.2022.09.017)
Supplement: Document S1. Figures S1–S — 4 [file mmc1.pdf]

OMTM, Volume *27*

Supplemental information

Intravenous immunoglobulin prevents peripheral liver transduction of intrathecally delivered AAV vectors

Makoto Horiuchi, Christian J. Hinderer, Jenny A. Greig, Cecilia Dyer, Elizabeth L. Buza, Peter Bell, Jessica A. Chichester, Peter M. Hayashi, Hanying Yan, Tamara Goode, and James M. Wilson

Supplemental Data

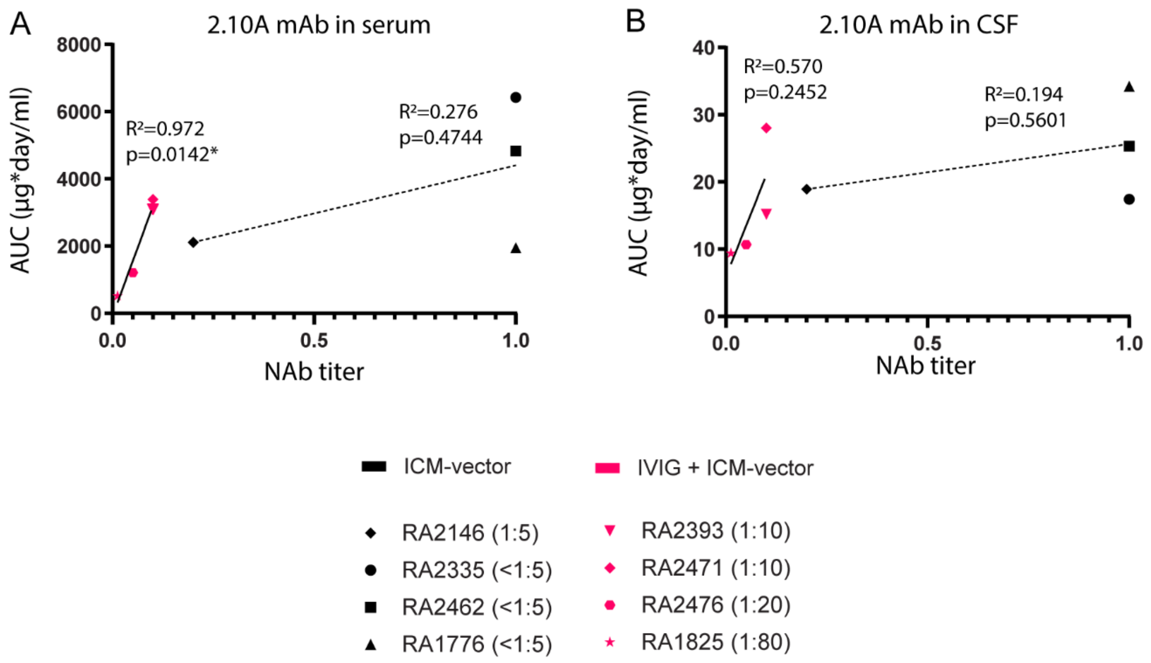

Figure S1. AUC of 2.10A rhIgG1 expression in serum (A) and CFS (B) along the NAb titer at day 0 as decimal numbers, where 1 indicates NAb titers below detection, <1:5. The dotted and solid lines are linear regression lines for the ICM-vector and IVIG + ICM-vector groups, respectively. R^2 values and non-zero p-values from linear regression analysis are shown. * indicates $p<0.05$.

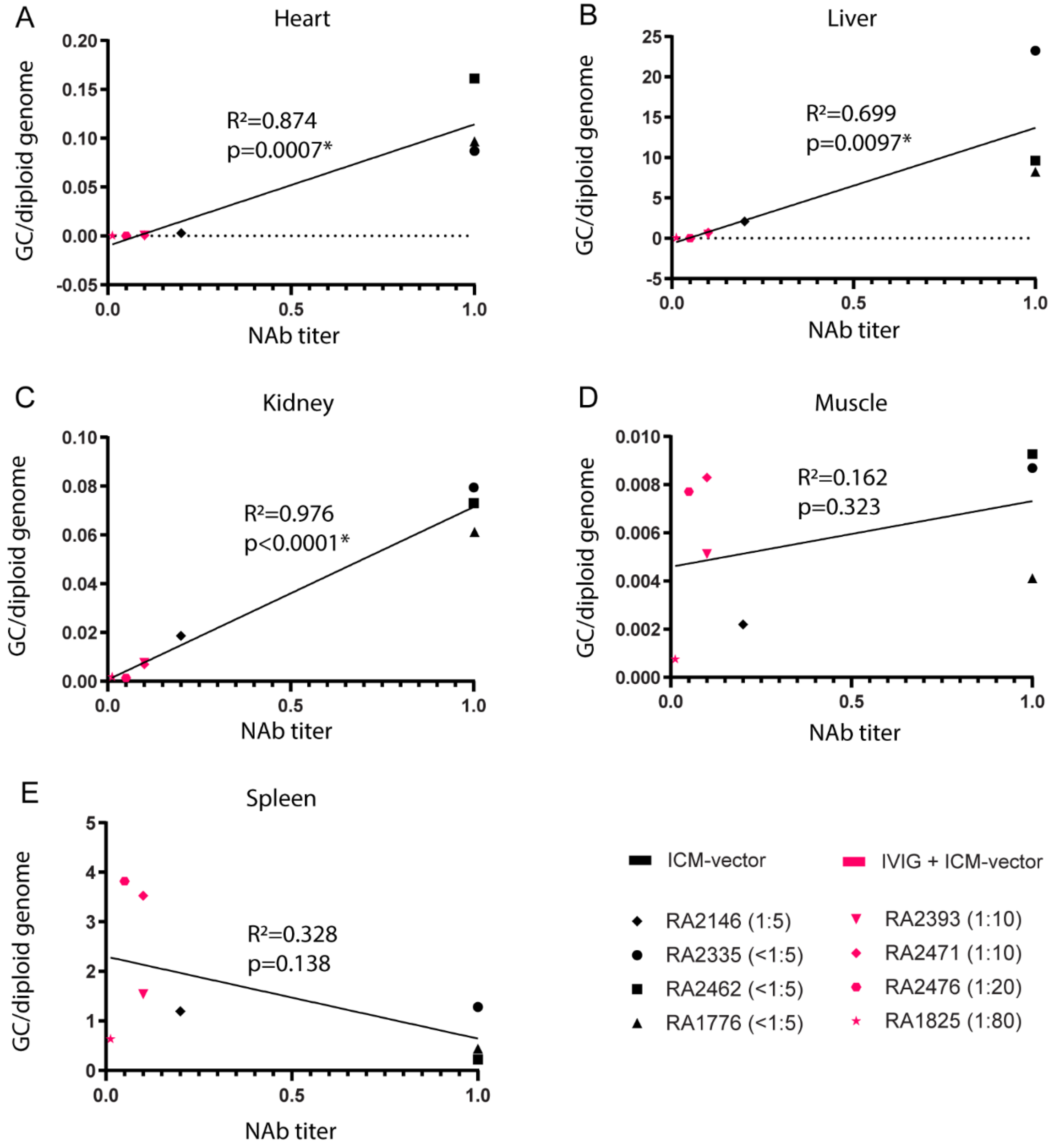

Figure S2. Vector genome biodistribution data for non-CNS tissues from individual NHPs. Tissue samples from necropsy at days 88–91 post-AAV administration were subjected to vector genome biodistribution qPCR analysis. Vector genome copy numbers (GC) for individual NHPs are shown along the NAb titer at day 0 as decimal numbers, where 1 indicates NAb titers below detection, <1:5. R^2 values and non-zero p-values from linear regression analysis are shown. * indicates $p<0.05$.

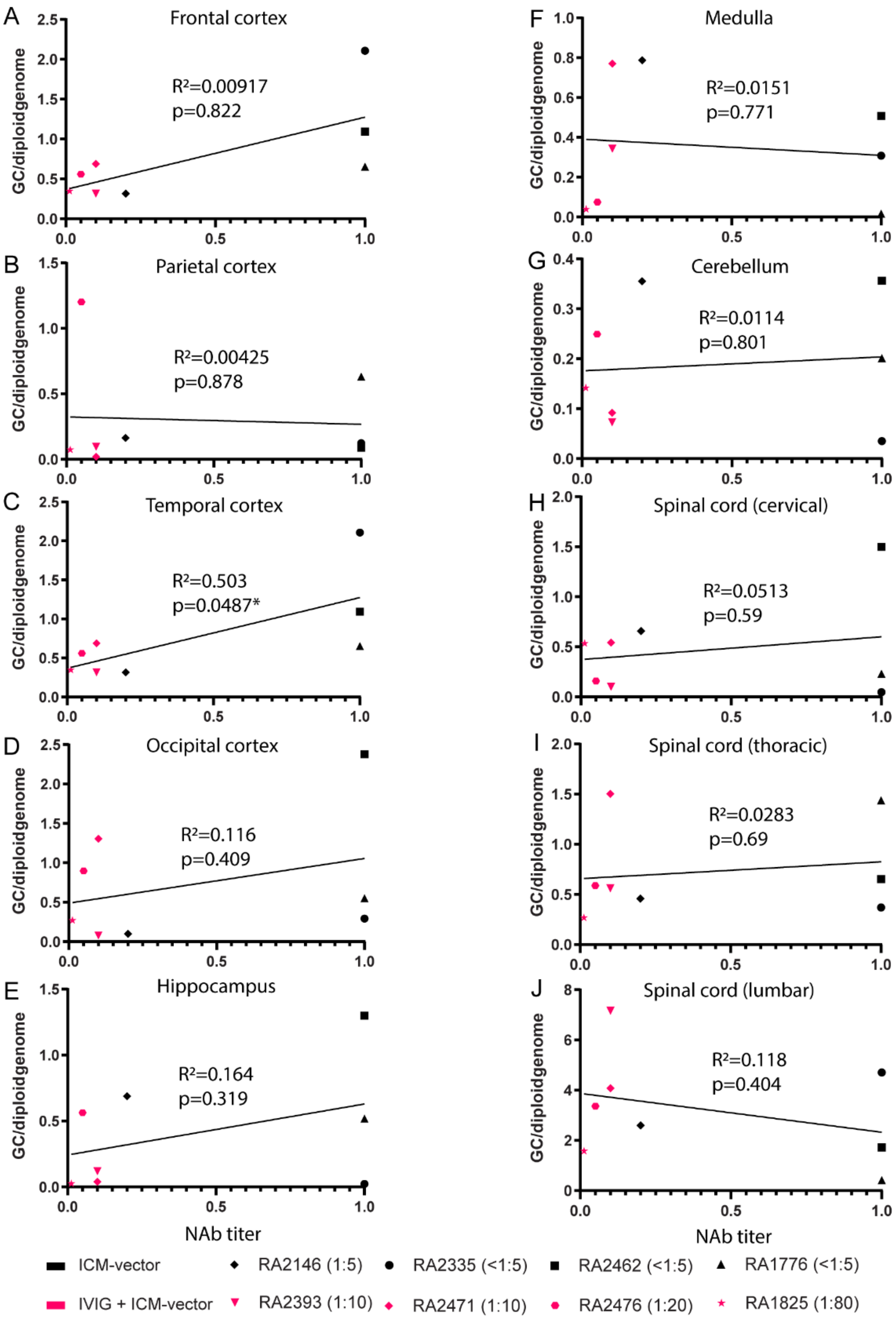

Figure S3. Vector genome biodistribution data for CNS tissues from individual NHPs. Tissue samples from necropsy at days 88–91 post-AAV administration were subjected to vector genome biodistribution qPCR analysis. Vector genome copy numbers (GC) for individual NHPs are shown along the NAb titer at day 0 as decimal numbers, where 1 indicates NAb titers below detection, <1:5. R^2 values and non-zero p-values from linear regression analyses are shown.

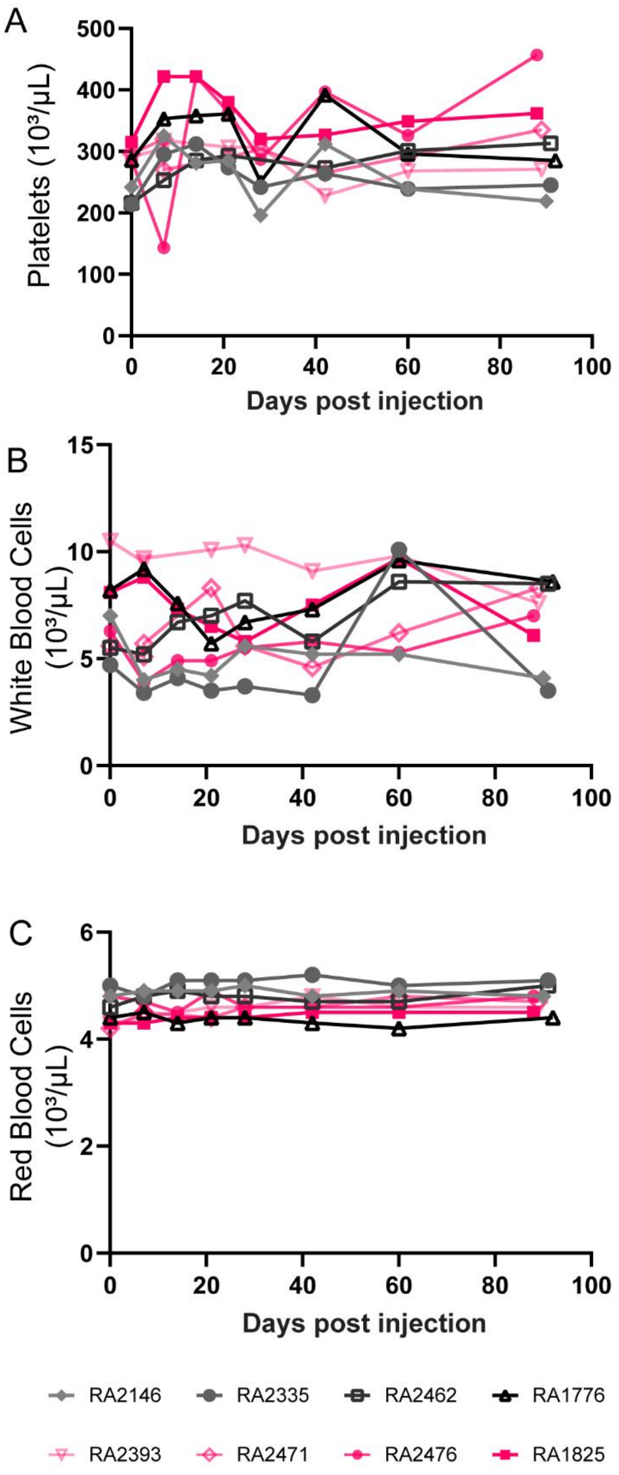

Figure S4. Blood cell counts of individual NHPs. Counts for platelets (A), white blood cells (B), and red blood cells (C) during the course of the study are shown.
